# Reporting the Presence of Coronary Artery Calcium in the Final Impression of Non-gated CT Chest Scans Increases the Appropriate Utilization of Statins

**DOI:** 10.7759/cureus.10579

**Published:** 2020-09-21

**Authors:** Raymond Fisher, Anthony Vandehei, Charles Haller, Joshua Boster, Brian Shipley, Christopher Kaatz, Jaclyn Harris, Satoshi R Shin, Lisa Townsend, Jessica Rouse, Sarah Davis, James Aden, Dustin Thomas

**Affiliations:** 1 Cardiology, Brooke Army Medical Center, Fort Sam Houston, USA; 2 Internal Medicine, Brooke Army Medical Center, Fort Sam Houston, USA; 3 Statistics, Brooke Army Medical Center, Fort Sam Houston, USA

**Keywords:** cac, statin, non-gated chest ct

## Abstract

Background

Coronary artery calcium (CAC) scoring based on gated non-contrast cardiac computed tomography (CT) is a validated risk marker of major adverse cardiovascular events (MACE). Reporting of CAC on non-gated CT chest (NGCT) scans and the impact on medical therapy is not well studied.

Methods

A retrospective cohort of 5,043 NGCT scans was reviewed for the presence of CAC. The radiology report was reviewed to determine whether CAC was mentioned in either the body of the report or the final impression. Electronic medical records (EMR) were abstracted for baseline demographics, cardiovascular (CV) risk factors, lipid-lowering agents, and aspirin (ASA) prior to and after NGCT.

Results

CAC was present in 63.0% of NGCT scans. Of these scans, CAC was mentioned in the body of the report in 81.6% of studies. Conversely, CAC was mentioned in the final impressions in only 15.1% of these scans. Amongst patients with CAC, initiation of a statin in treatment-naive patients was more common when CAC was mentioned in the final impression versus the body only (12.3% vs. 4.9%, p=0.001) despite the fact that baseline utilization of statins in this cohort was higher (71.1% vs. 64.1%, p=0.005). Initiation of a statin in treatment-naive patients had a trend towards significance when CAC was mentioned in the body of the report versus not reported (4.9% vs. 2.62%, p=0.142). Reporting of CAC in the final impression significantly increased the initiation of ASA in treatment-naive patients (9.52% vs. 4.33%, p=0.033). Reporting of CAC in either the final impression or the body of the report did not affect the initiation of non-statin lipid-lowering therapies in patients with CAC.

Conclusion

The inclusion of CAC in the final impression of NGCT radiology reports positively impacts the appropriate initiation of statin and aspirin therapy in treatment-naive patients. Universal adherence to a standardized reporting system for the presence of CAC on NGCT should be considered to improve the initiation of guideline-directed medical therapy.

## Introduction

Coronary artery calcium (CAC) is a well-validated risk marker of coronary artery disease (CAD), adding significant predictive power over traditional risk assessment factors and tools such as carotid media intimal thickness [1-4]. CAC scoring is traditionally performed utilizing electrocardiogram (ECG) gating with standard reconstruction and acquisition parameters (2-3 mm slice thickness and 120 kVp tube voltage) as described by Agatston and colleagues [5]. A plethora of data supports a strong relationship between the presence and extent of coronary calcification and clinical outcomes. This is demonstrated among multiple patient cohorts with various risk factors and ethnicities [6]. 

The US Preventive Services Task Force (USPSTF) released a recommendation for low-dose computed tomographic (CT) lung cancer screening for high-risk current and former smokers [7]. Additionally, under the Affordable Care Act, this level of recommendation carries with it a requirement for commercial insurers to fully cover low-dose CT screening. Thus, an estimated 7-10 million additional patients are anticipated to undergo chest CT screening [8]. As many of these patients fall into the intermediate atherosclerotic cardiovascular disease (ASCVD) risk category, these non-gated chest CT (NGCT) scans provide an opportunity to perform additional cardiovascular (CV) risk stratification as part of their lung cancer screening.

The practice of reporting CAC on non-gated chest CT scans is variable and non-standardized despite recent coronary artery calcium data and reporting system (CAC-DRS) guidelines that have formally recommended a method to quantify CAC on NGCT through visual estimation [9]. The routine reporting and characterization of CAC on NGCT scans may effectively communicate to the referring provider a patient's risk for future cardiovascular events for which preventative therapy and lifestyle modification can be aggressively pursued. Recent data has been published demonstrating a good correlation between CAC scores from non-gated chest CT scans and formal CAC testing [10-12]. Furthermore, multiple small investigations have reported an increased incidence of adverse cardiovascular events and death in patients with qualitative coronary calcification on non-contrast non-gated CT chest scans [13-17].

We sought to define an association between reporting the presence of CAC on NGCT within the study report on preventive medication interventions.

## Materials and methods

Study population

**Figure 1:**
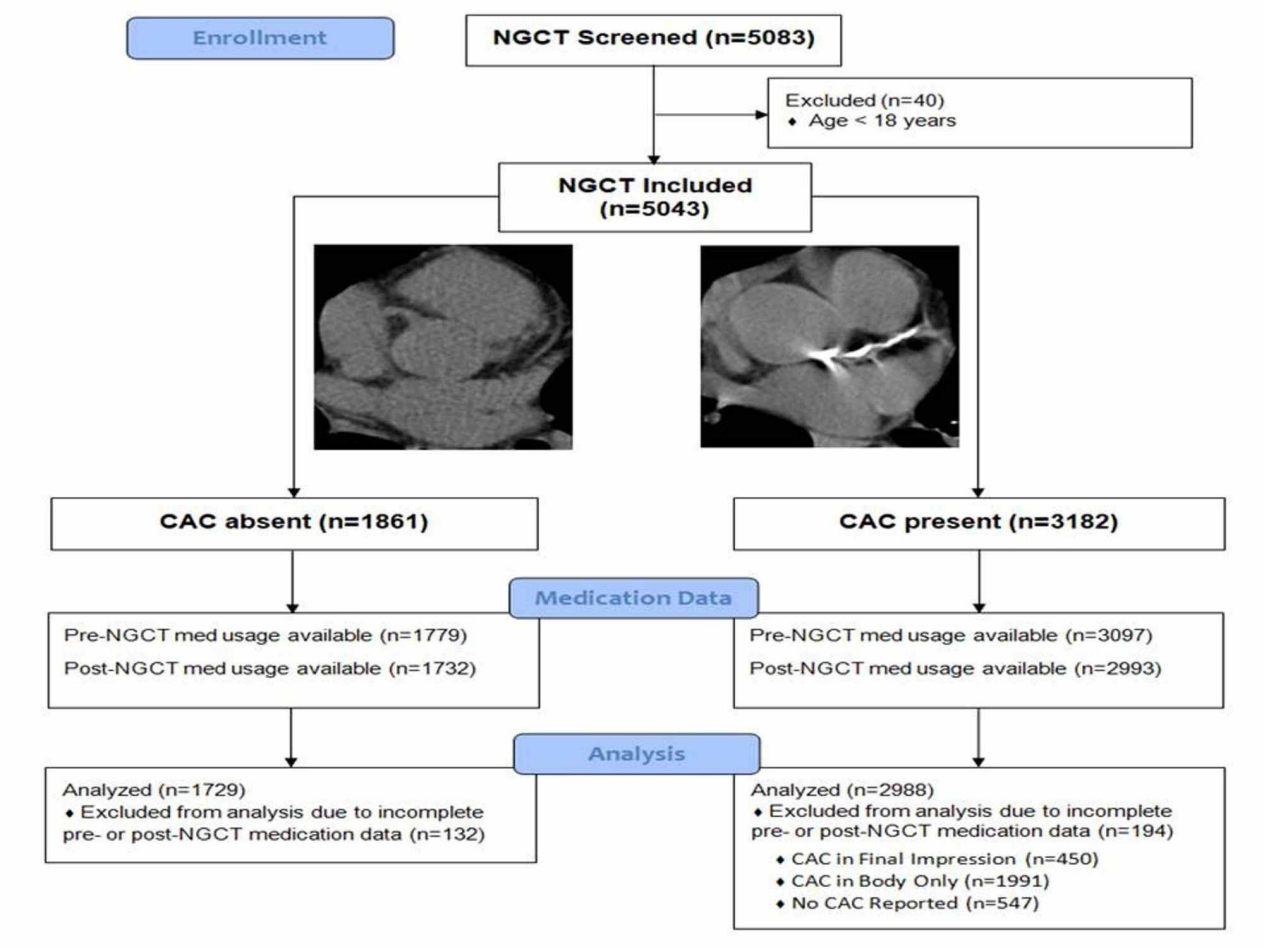
Study cohort flow diagram Enrolled 5,083 NGCT performed at a single institution over an 18-month period excluding those who were under the age of 18 at the time of the NGCT. The NGCT were qualitatively analyzed for the presence or absence of CAC. EMRs were reviewed to determine the pharmacotherapy prior to NGCT and subsequent initiation after NGCT. Subjects without records indicating pharmacotherapy prior to NGCT or subsequent initiation after NGCT were excluded. The remaining cohort was analyzed. NGCT - non-gated CT; CCTA - coronary CT angiography; CAC - coronary artery calcium

This is a single-center retrospective observational cohort study of patients who underwent non-contrast, non-gated chest CT (NGCT) scans between 1 January 2011 and 30 June 2012 through a picture archiving and communication system (PACS) query. Patients over the age of 18 years old at the time of the scan were included (Figure 1). These scans were reviewed qualitatively, by trained cardiology fellows/attendings, for the presence or absence of coronary calcification. Cardiovascular risk factors were abstracted via an electronic medical record (EMR) query. Hypertension was defined as a diagnosis of hypertension, within the EMR, or utilization of an antihypertensive medication prior to NGCT. Hyperlipidemia was defined as a fasting low-density lipoprotein level (LDL) > 190 mg/dL, a diagnosis of dyslipidemia in the EMR, or treatment with lipid-lowering medication. Diabetes mellitus was defined as a hemoglobin A1c ≥ 6.5% or prescription of anti-hyperglycemic medications. Known coronary artery disease (CAD) was defined as a prior myocardial infarction (MI), percutaneous coronary intervention (PCI), or coronary artery bypass graft (CABG) surgery. Active smoking was adjudicated based on smoking status, as documented in provider encounters ±2 months from the date of NGCT.

NGCT report analysis

The body and final impression of the NGCT reports were reviewed in the PACS. A portion of the report was demarcated as reporting CAC if "coronary artery calcification", "coronary atherosclerosis", "coronary vascular calcification", "coronary artery disease", calcification reported in a named coronary artery distribution, or evidence of previous CABG or PCI was annotated. Reporting of aortic calcification, valvular calcification, unspecified vascular calcification, or unspecified atherosclerosis was not considered indicative of reporting CAC. Furthermore, if no mention of any of the above terminology, the study was considered reported without CAC.

Medication management

Baseline and post-NGCT medications were abstracted from the EMR. Lipid-lowering medications (excluding fish oil) and aspirin (ASA) ordered prior to the date of NGCT were considered pre-NGCT medications. With respect to lipid-lowering therapies, patients with the continuation of previously prescribed statin, non-statin, or combination therapy were considered to have continued their baseline regimen. New initiation of a statin or non-statin was defined as a new prescription in a treatment-naive patient. Escalation of statin therapy was defined as an increased dose of the baseline statin or an increase from a lower intensity to higher intensity statin.

Statistical analysis

Continuous variables were analyzed utilizing two-sided Chi-squared testing. Comparison of means was performed using analysis of variance (ANOVA). A comparison of non-normally distributed continuous variables was reported as medians with inter-quartile ranges and analyzed using the Mann-Whitney test. Kaplan-Meier analysis was performed for event-free survival. Inter-scan and non-gated vs. gated CAC scan agreement was assessed using intra-class correlation (ICC) and Fleiss' Kappa (k).

## Results

Prevalence of CAC on NGCT

A total of 4,953 NGCT scans were reviewed. Overall, CAC was present in 63% of NGCT. Patients with CAC were twenty-three years older, over twice as likely to smoke or have type II diabetes mellitus, four times as likely to have hyperlipidemia, and five times as likely to have hypertension or known CAD. The baseline demographics and odds ratios for predicting CAC are shown in (Table 1).

**Table 1:** Comparison of demographics and pharmaceutical therapy for patients with CAC and without CAC at time of non-gated chest CT CAC - coronary artery calcium; OR - odds ratio; CI - confidence interval; DM2 - diabetes mellitus type 2; HTN - hypertension; HLP - hyperlipidemia; CAD - coronary artery disease; CT - computed tomography

	CAC (n=3119)	No CAC (n=1834)	OR predicting CAC with 95% CI	p-value
Age (years)	71±11	48±17		<0.0001
Male gender	1793 (57.5%)	891 (48.6%)	1.43 (1.27-1.61)	<0.0001
Smoker	1346 (43.2%)	493 (26.9%)	2.07 (1.82-2.34)	<0.0001
DM2	938 (30.1%)	252 (13.7%)	2.70 (2.32-3.15)	<0.0001
HTN	2578 (82.7%)	839 (45.7%)	5.65 (4.96-6.44)	<0.0001
HLP	2321 (74.4%)	704 (38.4%)	4.67 (4.12-5.28)	<0.0001
Known CAD	1148 (36.8%)	178 (9.7%)	5.42 (4.57-6.42)	<0.0001
Pre-CT statin	1960 (62.8%)	440 (24.0%)	5.18 (4.55-5.90)	<0.0001
Pre-CT nonstatin	451 (14.5%)	106 (5.8%)	2.76 (2.21-3.43)	<0.0001
Pre-CT aspirin	1827 (58.6%)	433 (23.6%)	4.58 (4.02-5.21)	<0.0001

Impact of reporting in CAC+ patients

The patient population was further characterized by the utilization of cardioprotective pharmacotherapy prior to and subsequent to the NGCT and how CAC was mentioned in the radiology report. The subcategorization of the patients, as well as reasons patients were excluded from the study, is provided in (Figure 1).

**Figure 2:**
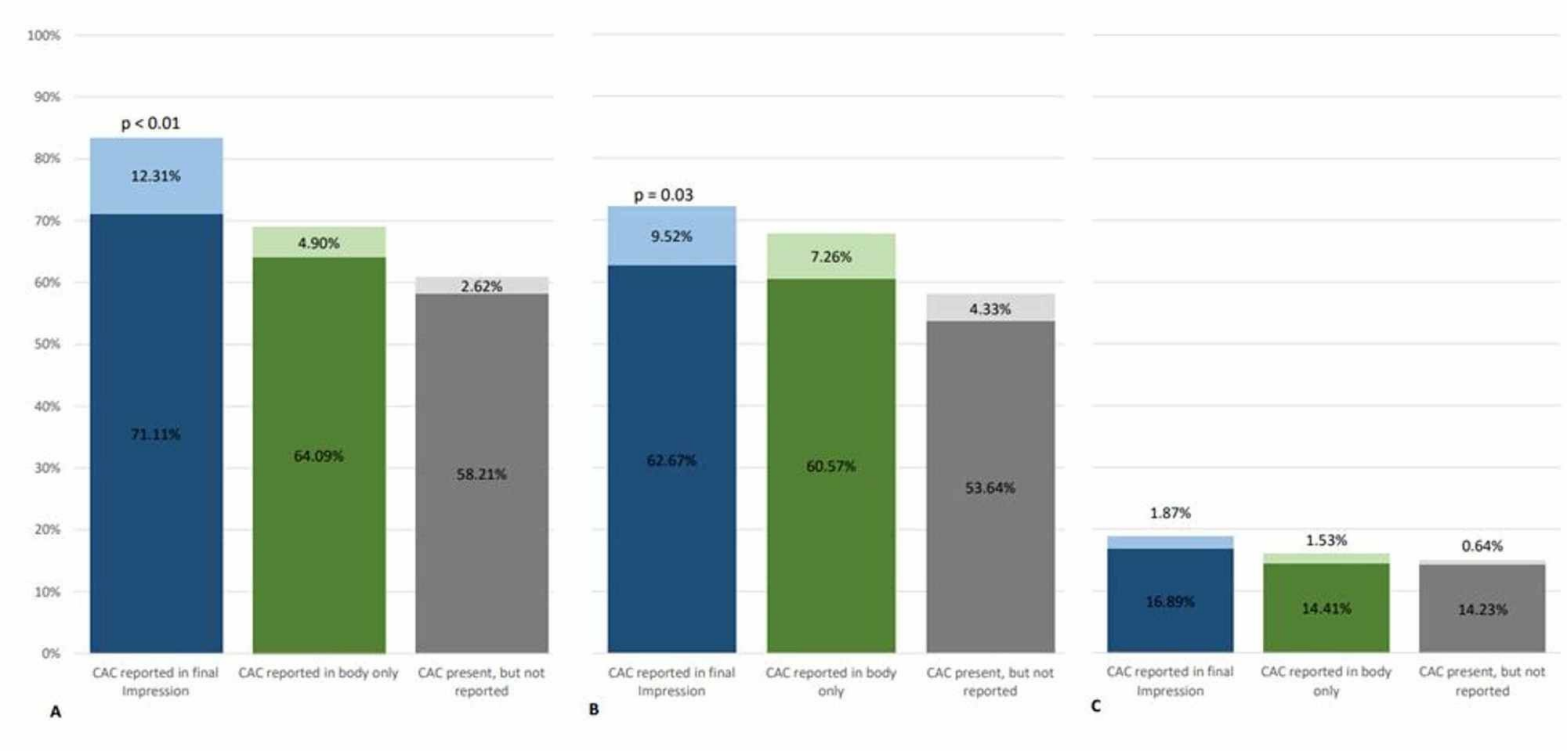
Composite graph illustrating pharmacotherapy prior to NGCT and subsequent initiation after NGCT The dark color represents baseline pharmacotherapy use prior to NGCT and the lighter color represents subsequent initiation of pharmacotherapy after NGCT. There was a statistically significant increase in the appropriate initiation of statin and aspirin therapy when the CAC was mentioned in the final impression of the radiology report. Panel A: graphical representation of baseline statin usage prior to NGCT and subsequent initiation after NGCT. Panel B: graphical representation of baseline aspirin usage prior to NGCT and subsequent initiation after NGCT. Panel C: graphical representation of baseline non-statin lipid lower agent prior to NGCT and subsequent initiation after NGCT. NGCT - non-gated CT; CAC - coronary artery calcium

Baseline utilization of statins in patients with CAC reported in the final impression was higher than those with CAC reported only in the body of the report (71.1% vs. 64.1%, p=0.005). Baseline utilization of ASA did not differ between those patients with CAC reported in the final impressions versus those with CAC reported in the body only.

Baseline utilization of statins in patients with CAC reported in the final impression and with CAC reported in the body only was higher than those with CAC that was not reported (71.1% vs. 58.2% and 64.1% vs, 58.2%, respectively; p≤0.01). Baseline utilization of ASA was higher in patients with CAC reported in the final impression and with CAC reported in the body of the report than in those with CAC that was not reported (62.6% vs. 53.6% and 60.5% vs. 53.6%, respectively; p<0.01).

Baseline utilization of statin, ASA, and non-statin lipid-lowering agents were higher in the group with CAC than in those without CAC (64.1% vs. 24.5%, 59.6% vs. 24.1%, 14.8% vs. 6.0%, respectively; p<0.001).

A composite graph illustrating pharmacotherapy prior to NGCT and subsequent initiation of pharmacotherapy is illustrated in (Figure 2).

Appropriate initiation of statin therapy in treatment-naïve patients was more common when CAC was mentioned in the final impression than in only the body of the radiology report (12.3% vs. 4.9%, p=0.001) despite the baseline utilization of statin therapy in this cohort being higher (71.1% vs. 64.1%, p=0.005). Initiation of statin therapy in treatment-naïve patients had a trend towards significance when CAC was mentioned in only the body of the radiology report versus when CAC was present and not mentioned (4.90% vs. 2.62%, p=0.142).

Appropriate initiation of ASA therapy in treatment-naïve patients was more common when CAC was mentioned in the final impression than when CAC was present and not reported (9.52% vs. 4.33%, p=0.033). Initiation of ASA therapy in treatment-naïve patients had a trend towards significance when CAC was mentioned in only the body of the radiology report versus when CAC was present and not reported (7.26% vs. 4.33%, p=0.101).

Reporting of CAC did not affect the initiation of non-statin lipid-lowering medications.

## Discussion

This is the largest retrospective cohort to analyze observed rates of medical therapy initiation and continuation based on the reporting of CAC on NGCT. In this study, patients with CAC on NGCT had that finding included in the final impression in only 15.1% of total scans with CAC. However, among patients with CAC, those with it reported in the final impression had a statistically significant improvement in the initiation of appropriate aspirin and statin therapies despite having a statistically significant higher baseline utilization of these medications. This statistically significant increase in the initiation of statin and aspirin therapy was not seen in the larger cohort of patients in which CAC was only mentioned in the body of the radiology report.

There is a growing wealth of data correlating CAC on NGCT with formal CAC scoring and higher adverse cardiovascular events. The availability of patient-specific risk assessment through CAC on NGCT will expand significantly as a result of the recent recommendation by the US Preventive Services Task Force for the screening for lung cancer with low dose NGCT. It is estimated that up to 10 million patients would qualify for this screening and for whom concomitant cardiovascular risk stratification with CAC can be performed. However, like any other diagnostic test, the impact on patient outcomes requires effective communication of important test findings to the referring provider. Currently, the practice of reporting CAC on non-gated CT chest scans is variable. This is in stark contrast to Breast Imaging, Reporting and Data System (BI-RADS) for mammograms and Coronary Artery Disease Reporting and Data System (CAD-RADS) for gated coronary studies, which provide a standardized means of reporting findings to the ordering providers. There are examples of standardized reporting recommendations that have effectively standardized downstream evaluation and management with subsequent improvement in patient outcomes and quality. For example, Liver Reporting and Data System (LI-RADS) provides a standardized reporting protocol for evaluation of liver lesions effectively communicating to providers the necessity of liver biopsy and continued eligibility for liver transplantation [18]. Similarly, CAD-RADS provides recommendations based on coronary atherosclerotic lesion characteristics indicating whether further testing is required or evaluation for the non-cardiac origin of symptoms and reassurance is appropriate [19]. Based on these findings, universal adherence to a standardized report system, such as that outlined in CAC-DRS, with reporting in the final impression including recommendations for ordering providers, would have a significant impact on the initiation of guideline-directed medical therapy.

Limitations of this trial include its retrospective design, which hinders our ability to assign causality. For example, medication changes noted may not have been a result of the NGCT. This study represents a closed referral, single-center analysis; therefore, its conclusions may not be generalizable to other practice settings. Additionally, this study design was unable to determine the impact of confounders on the initiation of statin or ASA therapy, including bleeding concerns, previous intolerances, or patient preference. The addition of quantitative or semi-quantitative analysis of the NGCT, such as calculating non-gated Agatston scoring or ordinal scoring, could further expound on this data and the effect of medication interventions on this population.

## Conclusions

Inclusion of CAC in the final impression of NGCT radiology reports positively impacts the appropriate initiation of statin and aspirin therapy in treatment-naïve patients. Universal adherence to a standardized reporting system for the presence of CAC on NGCT should be considered to improve the initiation of guideline directed medical therapy. 
